# Food Knowledge for Better Nutrition and Health: A Study among University Students in Portugal

**DOI:** 10.3390/healthcare11111597

**Published:** 2023-05-30

**Authors:** Raquel P. F. Guiné, Sofia G. Florença, Maria Graça Aparício, Ana Paula Cardoso, Manuela Ferreira

**Affiliations:** 1 CERNAS Research Centre, Polytechnic Institute of Viseu, 3504-510 Viseu, Portugal; 2 UICISA:E Research Centre, Polytechnic Institute of Viseu, 3504-510 Viseu, Portugal; 3 CIDEI Research Centre, Polytechnic Institute of Viseu, 3504-510 Viseu, Portugal

**Keywords:** food literacy, knowledge, nutrition, health, food consumption, questionnaire survey

## Abstract

When students enter university, they suffer adaptations, including, usually, greater autonomy and responsibility for the choices they make. Therefore, it is crucial that they are well informed so as to make healthier food choices. The aim of this study was to determine whether sociodemographic characteristics, academic performance and lifestyle (tobacco and alcohol consumption) interfere with food literacy in university students. A quantitative, analytical, descriptive, transversal and correlational study was carried out, using quantitative data obtained through a questionnaire survey applied to a sample of 924 university students in Portugal. Food literacy was assessed through a scale of 27 items, distributed in three dimensions: D1—Literacy about food nutritional value and composition, D2—Literacy about labelling and food choice and D3—Literacy about healthy eating practices. Results showed no differences in food literacy according to sex or age. However, food literacy varied significantly with nationality, either globally (*p* = 0.006) or in the different dimensions evaluated (*p*-values of 0.005, 0.027 and 0.012 for D1, D2 and D3, respectively). In terms of academic achievement, the results showed no significant differences according to self-reported academic performance or even to the average classification obtained in the course. Regarding lifestyle variables, it was observed that alcohol consumption or smoking are not associated with food literacy, that is, food literacy does not vary significantly with these two lifestyle variables. In conclusion, food literacy in general and the dimensions evaluated are essentially constant among university students in Portugal, only varying for students from abroad. These results help to better perceive the food literacy levels for the population under study, university students, and that can be a valuable tool to better increase food literacy at these institutions as a way to better prepare for a healthier life and proper food habits that can enhance health in the long term.

## 1. Introduction

Food is a decisive factor for the health of individuals, as it has a direct influence on both physical and psychological well-being. Healthy eating presupposes a diet that is complete, varied, balanced and provides adequate calories. The average energy values obtained through food for healthy adults vary between 1800 and 2500 calories, depending on the lifestyle of each individual [1,2,3,4].

Despite all the existing information for the promotion of healthy eating, sometimes it is impossible for families of lower income to carry out this type of diet, which leads to a direct negative impact on eating patterns [5,6]. More economically developed societies have access to a greater diversity of foods, which makes it easier for them to adopt better food choices. However, sometimes this availability of food can lead to overfeeding, which, when associated with other factors, such as reduced time to prepare meals, an increase in meals eaten outside the home and an increase in the supply of energy-dense foods, can have harmful consequences for the health of populations. In recent decades, there have been more and more changes in the eating habits of populations, which have led to an increase in the prevalence of overweight in more developed countries [7,8,9,10,11,12,13,14].

Adequate eating habits are crucial for the health status of individuals, mainly in preventing the incidence of chronic non-communicable diseases, such as cardiovascular diseases [15,16,17]. It is known that most existing diseases are attributable to unhealthy eating habits acquired since childhood [18,19]. The WHO states that a correct diet is part of a healthy lifestyle and that it would prevent 90% of registered cases of type 2 diabetes mellitus and 80% of cardiovascular diseases [20].

According to the Global Burden of Disease (GBD) study in 2017 [21], inadequate eating habits of the Portuguese were the third risk factor that contributed most to the loss of years of healthy life. Excessive consumption of fats and salt and insufficient consumption of vegetables, fruits, oilseeds and cereals are the main inappropriate eating behaviours. Excessive salt consumption in Portugal is one of the greatest risks to public health, thus becoming a topic of interest for health professionals who must propose measures to reduce its consumption [22]. If these measures are effective, they will benefit the health of populations, particularly when it comes to cardiovascular diseases. The recommended replacement of salt by aromatic herbs can be a good choice, adding colour, flavour and aroma to dishes, and contributing to the ingestion of important micronutrients such as vitamins (A, B and C) or minerals (calcium, phosphorus, potassium and iron) [23,24].

Food choices and habits are influenced by several factors, including economic, psychological, social and cultural, as well as knowledge about food and nutrition. There are several studies that show that the more in-depth the knowledge about food and nutrition, the easier it is to make healthier food choices [25,26]. The main means of providing information range from health professionals, family and friends to the internet and other different means of communication [27,28,29]. The various means of communication play an important role in promoting healthy habits as they allow easy access to health information. However, incorrect or contradictory information is often available, which can mislead individuals about their habits and choices [25]. Cline and Haynes [30] alert that the Internet is increasingly a privileged source of information, with hundreds of websites about health information, much of which is inaccurate, liable to cause confusion or even dangerous. Access to information, especially correct information, is very important for health literacy and, consequently, for the practice of healthy eating [31].

The entry of students into higher education implies adaptations and entails greater autonomy and responsibility for the choices made. Therefore, it is essential that students have literacy in several areas, namely in what refers to food and nutrition. Higher education students can be considered a risk group for having inappropriate lifestyles, given that the transition from secondary education to university represents a period of freedom and independence. It is essential that university students have or acquire food literacy so that they are able to look for relevant information as well as understand and use it properly to have healthy behaviours. Therefore, the objectives of this study were to determine whether sociodemographic and academic variables interfere with food literacy and to analyse how lifestyle influences food literacy in university students.

## 2. Materials and Methods

### 2.1. Research Questions

This research was built around three main research questions, as follows:

RQ1: How do the sociodemographic variables interfere with food literacy among university students?
RQ2: What is the influence of academic variables on food literacy among university students?
RQ3: How do contextual variables relate to lifestyle impact food literacy among university students?

**Figure 1:**
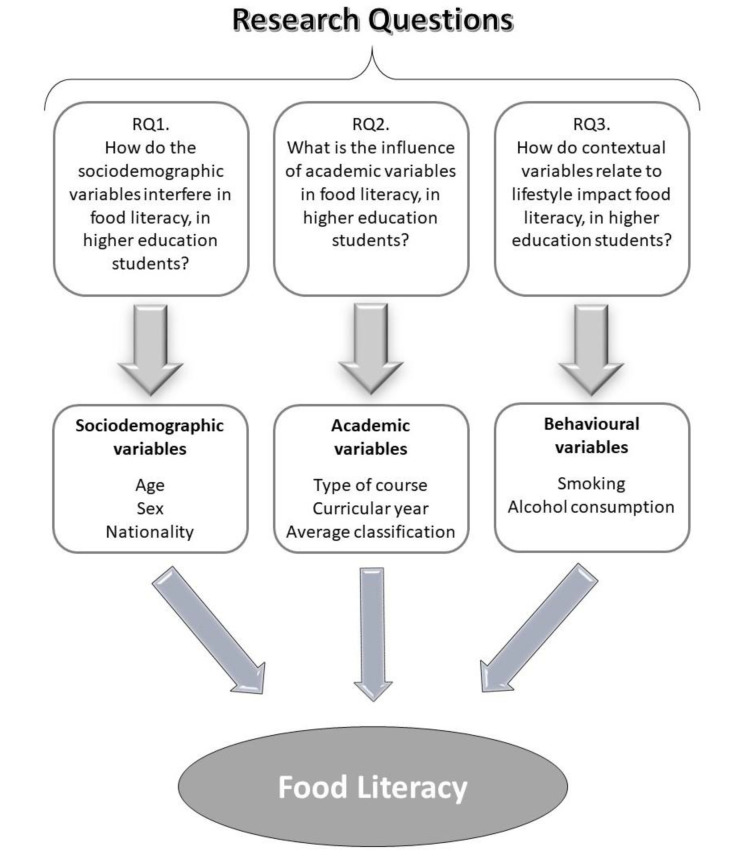
Schematic representation of the relationship between the variables studied in the empirical investigation and the research questions.

The graphical representation of the problem modelling is shown in Figure 1.

### 2.2. Investigation

To answer the questions and attain the proposed objectives, we carried out a quantitative, analytical, descriptive, transversal and correlational study. The questionnaire consisted of different sections (Appendix A), intended to evaluate sociodemographic characteristics, habits related to smoking and alcoholic beverage consumption, academic variables, and a number of items aimed at evaluating food literacy levels, to be answered on a scale with 4 points for rank of difficulty complemented with an option of ‘I don’t know’. The validation of the scale is described in Guiné et al. [32]. To carry out the present study, data were collected through the application of questionnaires (completed online, voluntarily and anonymously) from January to April 2021, in the academic year 2020/2021.

During the preparation of this study, ethical standards were respected. Participants were informed of the anonymity and confidentiality of the data they provided for the study, as well as its purpose, and the questionnaires were filled out voluntarily. Authorization n° 15/SUB/2020 was granted from the Ethics Committee of the Instituto Politécnico de Viseu for the application of questionnaires and data collection.

### 2.3. Sampling

In this study, we used a non-probabilistic sample composed of 924 university students. The subjects were selected using a convenience methodology, in which some classes were selected to apply the questionnaire. These selected classes included different courses, in different faculties and different course years. Additionally, the different courses were variable in the level of education, varying from CTESP to master’s courses. CTESP, or Higher Technical Professional Courses, are 2-year courses offered at Portuguese universities aimed at rapid professionalization. These courses are level 5 in the Qualifications framework. Additionally, bachelor courses (level 6) and post-graduate and master courses (level 7) were selected for the research in various scientific domains, including health sciences, agrifood sciences, educational sciences, technology and engineering courses, tourism, accounting and finance. For the selected classes, the teachers facilitated the data collection and used part of the class to let the students fill out the questionnaire as a way to get full participation from the students in each of the classes. For that reason, the number of invitations and the number of questionnaires responded to were even, in contrast with what could have happened if the students were asked to fill out the questionnaire in their free time. The sample included students from Higher Education schools in the central region of Portugal.

### 2.4. Data Analysis

For data analysis, descriptive statistics and analytical or inferential statistics were used. The use of descriptive statistics made it possible to determine the absolute and percentage frequencies; some measures of central tendency, namely, the averages and measures of dispersion such as the amplitude of variation, the coefficient of variation and the standard deviation, were also used; and finally, measures of shape such as asymmetry (skewness) or flatness (kurtosis). When interpreting the coefficient of variation, the following reference values are used. The reference values for the coefficient of variation are 0% to 15%—low, 16% to 30%—moderate and above 30%—high [33,34].

With regard to inferential statistics, parametric and non-parametric tests were used, namely,

⮚ Chi-square test: a non-parametric test that assesses the relationship between two qualitative variables;
⮚ Adjusted residuals—coupled to chi-square: used to locate existing differences in Chi-square test;
⮚ Mann–Whitney U test: a non-parametric test used for independent samples to compare the means of a quantitative variable in two groups;
⮚ One-way analysis of variance (ANOVA): a parametric test that allows comparing the means of a quantitative variable in three or more independent samples and, thus, telling us if there are differences between the groups;
⮚ Post hoc test—coupled to ANOVA: used to locate existing differences in ANOVA;
⮚ Kruskal–Wallis test: a non-parametric test that allows comparing several independent samples from three or more groups.

A level of significance of 5% was considered in all statistical analyses, which were performed using SPSS (version 28) software.

## 3. Results

### 3.1. Sample Characterization

#### 3.1.1. Sociodemographic Characteristics

With regard to the age of university students in the sample, the minimum age was 18 years and the maximum was 70 years, with an average age of 22.35 years (±6.10 years). For women, the minimum and maximum ages ranged from 18 to 57 years, while for men, the ages ranged from 18 to 70 years. Women, on average, were slightly younger than men, with an average of 21.89 years (±5.37 years), while men had an average age of 24.14 years (±8.17 years). The coefficients of variation indicate a moderate dispersion (24.53%) for women and a high dispersion for men (33.84%).

It was verified that the female sex represents the majority of the sample, 79.7% (n = 736), while the male sex represents 20.3% (n = 188).

Regarding age, the group aged 22 years or older corresponded to 34.2% of the sample, of which 24.9% were female and 9.3% were male. The group aged less than or equal to 19 years old represented 33.9% of the sample, of which 28.8% were female and 5.1% were male. With regard to the group aged between 20 and 21 years inclusive, it represented 32% of the sample, of which 26.0% corresponded to females and 6.0% to males.

With regard to nationality, 96% of the students were Portuguese, with 77.3% females and 18.7% males, while 4.0% of the sample was of foreign nationality, of which 2.4% were females and 1.6% were males.

#### 3.1.2. Academic Variables and Performance

Concerning the type of course attended, 75% of the sample attended a bachelor’s degree, 62.1% females and 12.9% males. On the other hand, 17.4% attended a master’s degree, including 13.3% females and 4.1% males. Only 2.1% of the sample attended a Higher Technical Professional Course (CTESP), of which 0.5% were females while 1.5% were males. Finally, 5.5% of the sample attended other courses.

It was further observed that 40.6% of the sample attended the first year of the course curriculum, including 32.8% females and 7.8% males. The second curricular year represented 28.7% of the sample, with 23.2% females and 5.5% males. The third curricular year represented 20.1% of the sample, with 14.7% females and 5.5% males. The fourth curricular year corresponded to 8.9% of the sample, including 7.5% women and 1.3% men. Finally, the fifth curricular year represented 1.8%, with 1.4% women and 0.3% men.

When analysing the academic performance, we found that 60.6% of the sample considered that they had a good academic performance, with 48.8% females and 11.8% males, while 18.8% considered themselves to have a very good academic performance, with 15.8% females and 3.9% males. On the other hand, 17.7% of the sample was considered to have reasonable academic performance, including 13.4% females and 4.3% males. Still, 1.9% of the sample was considered to have excellent performance, with 1.3% females and 0.6% males. Finally, 0.9% of the sample claimed to have poor academic performance, with 0.3% being female and 0.5% male.

With respect to the average classification on a scale from 0 to 10 points, where 10 points is the threshold to pass, 60.2% had a score between 14 and 16 points (50.6% for females and 9.6% for males), and 29.3% had a classification in the range 10–13 points (21.2% females and 8.1% males). A smaller fraction had very high classifications (in the range from 17 to 20 points), mostly females (7.7% against 2.3% males), and only 0.5% had an average classification below 10 points (0.3% females and 0.2% males).

### 3.2. Lifestyle—Smoking and Alcohol Consumption

The contextual variables of lifestyle (consumption of tobacco and alcohol) were analysed according to the sex of the study participants, as indicated in Table 1. The students were asked if they had ever smoked tobacco, and it was found that the majority reported never having smoked (74.6%), of which 60.3% were female and 14.3% male. On the other hand, 12.6% of the students said they currently smoke and 12.9% have already smoked. These results are in accordance with those from Alves et al. [35], who conducted a survey of students from a university in Portugal and reported that only 20.1% of them were smokers, of whom 7.3% were occasional smokers, 2.9% were regular smokers and 9.9% were daily smokers. Modern health trends towards smoke-free university campus policies find some barriers, and some students fail to respect those policies.

It was also sought to understand whether university students consumed alcohol, and the results showed that the majority answered yes (73.9%), 57.7% were female and 16.2% were male, while 8.8% of the students reported never having consumed alcohol, with a representation of 6.8% females and 1.9% males.

The results of the chi-square test (Table 1) indicated that sex had no effect on tobacco consumption, since the *p*-value of the chi-square test was greater than the significance considered of 0.05 (*p* = 0.170). Regarding alcohol consumption, there was a significant difference between sexes, that is, being male or female has an effect on alcohol consumption, since the chi-square test value of *p* is significant (*p* = 0.025). Adjusted residuals positive and higher than 2 allow perceiving where those differences are: ‘yes’ to alcohol consumption for males and ‘no’ for females.

### 3.3. Dimensions of Food Literacy

The items used to assess food literacy, 26 in total, are presented in detail in Appendix A, and were grouped according to three dimensions: (a) literacy about food nutritional value and composition; (b) literacy about labelling and food choice; (c) literacy about healthy eating practices. Each item was scored on a 5-point scale (1 = very difficult, 2 = difficult, 3 = easy, 4 = very easy, 5 = I don’t know). To this end, the answers corresponding to 5 (I don’t know) were excluded from the analysis. For each participant, three indices were calculated as the sum of all items’ scores in each of the dimensions, then expressed in percentage (for example, for dimension 1, with 10 items, a minimum sum of 10 and a maximum sum of 40 correspond to a minimum score of 25 and a maximum of 100). Additionally, an index was calculated for the sum of all items, accounting for global food literacy. The results are presented in Table 2. The global literacy index has a mean value of 80.22 points out of 100, which corresponds to a value expressing that, in general, the university students in the studied sample consider it very easy to get information about food and to understand it to make more informed food choices. Considering the three dimensions of food literacy, those about healthy eating practices (D3) and food composition and nutrition value (D1) have indices with a mean value higher than the global, meaning that these aspects are considered by the students as those for which it is easier to be informed. On the contrary, dimension D2, about labelling and food choice, has the lowest mean score (77.94), but is still high. In all cases, the coefficients of variation correspond to a moderate dispersion of the data. Data for skewness indicate that all distributions are asymmetrical, left-skewed. Regarding kurtosis, the distributions for D1 and D2 are mesokurtic, but the distribution for D3 is leptokurtic.

### 3.4. Association between Sociodemographic Variables and Food Literacy

The level of food literacy was defined as low, medium or high, depending on the score for each participant. This level of literacy was analysed for the university students in the sample as a function of sex, with results presented in Table 3. It is shown that 49.2% of the sample has a moderate level of literacy, with 39.1% female and 10.1% male. Although a large part of the sample has moderate levels of food literacy, there is still 25.6% of the sample with low levels of literacy, which is a relevant part (one fourth of the sample). Only 25.1% of the sample has a high level of literacy in the field of food, with 20.6% being female and 4.5% male. There are no statistically significant differences between sexes for food literacy levels (χ^2^ = 1.12; *p* = 0.571). When analysing the level of food literacy according to nationality (Table 3), we see that, both in Portuguese and foreign nationalities, the most frequent level of literacy is moderate (49.2%). There are highly significant statistical differences between food literacy level and nationality (χ^2^ = 13.352; *p* = 0.001). According to the adjusted residuals, differences exist between students from other nationalities with a low level of literacy and Portuguese students with a moderate level of literacy.

The Mann–Whitney test was performed to understand the relationship between food literacy and sex (Table 4). The results revealed that females are more literate about the nutritional composition of foods (D1) and healthy eating practices than males (D3). However, males are more literate in labelling and food choices (D2). Nevertheless, these small differences are not statistically significant, given that the values of *p* are all higher than 0.05. Regarding food literacy as a function of nationality, the Mann–Whitney test was also carried out (Table 4), which revealed that Portuguese students present higher average orders than foreign students for all dimensions of food literacy. These differences are statistically significant in all cases (*p* varying from 0.005 to 0.027).

To verify whether there were significant differences in food literacy between age groups of the university students, the ANOVA test was performed (Table 5). If there were significant differences (*p* < 0.05), the homogeneity tests (post hoc) would identify which groups differ from each other. However, that was not verified in the present case, since no statistically significant differences were found when relating age and food literacy in its different dimensions or globally, with p-values always higher than 0.05. This means that age, in this range, does not significantly influence the food literacy of university students. The age groups are all very close to each other since they cover the ages of the students attending university, so they are mostly very young adults.

Although the differences are not statistically significant, some trends could be observed from the values in Table 5. University students aged 22 and over are the ones with the highest literacy in the nutritional composition of foods (81.41 ± 14.87). On the other hand, students aged 19 years or younger showed lower literacy in the nutritional composition of foods (79.80 ± 3.96). When analysing literacy about labelling and food choices, the group aged 22 years or older stands out with more knowledge (79.13 ± 16.39). Regarding literacy about healthy eating practices, the age group between 20 and 21 years old stands out as the most literate (81.89 ± 14.19).

### 3.5. Association between Academic Performance and Food Literacy

The ANOVA test was carried out to verify if there were significant differences between the food literacy of university students attending different academic degrees. By analysing Table 6, it is possible to see that there are no significant differences between the food literacy of the students attending different academic degrees, as the p-value is always greater than 0.05, which is corroborated by the post hoc test (Tukey). Although not statistically significant, some trends can be pointed out, namely that the students attending a university graduation are those with greater literacy about the nutritional composition of foods (D1) (80.7 ± 14.6) and about healthy eating practices (D3) (82.2 ± 13.4). Students who are attending a bachelor’s, master’s or other degrees are the ones with the highest literacy on labelling and food choices (D2) (average of 78.0).

The results of the Chi-Square test presented in Table 7 allow us to verify that there is not a statistically significant relation between the self-reported academic performance and the level of food literacy, according to the value of *p* (0.113), which is greater than the significance level established (*p* < 0.05). The results further show that good academic performance is the most common, accounting for a total percentage of 60.6%, regardless of the food literacy level: 15.7% for low; 28.8% for medium and 16.1% for high food literacy levels.

The students’ self-reported academic performance is not significantly related to food literacy, either globally or in its different dimensions, based on the results of the Kruskal–Wallis tests presented in Table 8, since in all cases the values of p are equal to 0.05 or higher. Nevertheless, it is possible to conclude that the students with the highest literacy regarding the nutritional composition of foods (D1) are those who obtained a very good academic performance degree (OM = 495.11). These students also revealed the highest literacy about labelling and food choices (D2) (OM = 492.91). Students with excellent academic performance are those who are more literate about healthy eating practices (D3) (OM = 497.08).

Table 8 also presents the results of the Kruskal–Wallis test to verify if there is a relationship between food literacy and the average classification of the university students in the study sample. From the values of p, is concluded that food literacy does not vary significantly with the average classification that the students have in their course. We found that students with an average rating between 14 and 16 points stand out with greater literacy on the nutritional composition of foods (D1) (OM = 476.03). However, students with an average classification below 10 points are more literate about labelling and food choices (D2) (OM = 520.90), as well as about healthy eating practices (D3) (OM = 519.70).

### 3.6. Association between Lifestyle Variables and Food Literacy

In Table 9, the relation between the food literacy dimensions among university students and tobacco and alcohol consumption is analysed using the Kruskal–Wallis test. The results reveal that students who used to smoke are the ones who are less literate in aspects related to food in general and in the different dimensions analysed. On the contrary, the group of students who currently smoke presented the highest literacy about the nutritional composition of foods (D1) (OM = 493.13) and healthy eating practices (D3) (OM = 472.01). Students who never smoked are the most literate group about labelling and food choices (D2) (OM = 470.86). We can also see that the students with more literacy about food in general (OM = 479.34) are those who currently smoke. However, these differences observed are not statistically significant, as the p-values of the Kruskal–Wallis tests are greater than 0.05, and so tobacco consumption has no effect on the diet of university students.

Regarding the relationship between alcohol consumption and food literacy, the results of the Kruskal–Wallis tests are also presented in Table 9, and they reveal that the group with the lowest food literacy in all evaluated dimensions is the group of students who have never consumed alcohol. In contrast, the group with the highest literacy on the nutritional composition of foods (D1) (MO = 466.19) and on labelling and food choices (D2) (OM = 466.47) is the group of students who consume alcohol. Students who do not consume alcohol are the group with the highest literacy about healthy eating practices (D3) (OM = 466.62) and also with the highest global food literacy (OM = 466.63). However, alcohol consumption does not influence food literacy since the *p*-values of the Kruskal–Wallis test were greater than 0.05.

## 4. Discussion

The results of this work highlighted some trends in the food literacy of higher education students in Portugal, and how these vary according to sociodemographic or behavioural factors, especially those linked with alcohol or tobacco consumption. The results obtained indicated that from the sample under study, only a minor fraction are tobacco consumers (around 13% smoke presently and also about 13% quit smoking), against a vast majority who never smoked. These results are in accordance with those from Alves et al. [35], who conducted a survey of students from a university in Portugal and reported that only 20.1% of them were smokers, of whom 7.3% were occasional smokers, 2.9% were regular smokers and 9.9% were daily smokers. These results are a direct consequence of the very strict national policies in Portugal, which made it legally forbidden to smoke in closed public places, or even in open spaces as long as they were in educational facilities or nearby. Although it is forbidden to smoke, modern health trends towards smoke-free university campus policies find some barriers, and some students fail to respect those policies. According to Braverman et al. [36], those students who have nicotine dependence tend to violate the norms more easily, while respect for the tobacco-free policy was found to be positively associated with smoke-free campus support, age, estimates of student policy support and cigarette smoking, and self-reported absence of smoking impulses. A study with university students in Barcelona, Spain [37] revealed that they accepted indoor smoke-free policies in the university buildings, but extending smoke-free regulations to outdoor areas of the university campuses was more limited. Sabrian and Utomo [38] studied the perception of different players (students, lecturers and staff members) on the smoke-free campus policy, and concluded that interventions can be introduced to enhance support from the staff group; however, the majority of the students, lecturers and staff were very supportive of creating a smoke-free campus. These results, along with supporting literature, encourage the continuation of smoke-free policies as a way to discourage the students from smoking and therefore preserve their future health, especially that related to cancer associated with organs that are affected by smoke, such as lung or oropharyngeal cancers, which are responsible for many deaths worldwide and are proven to be associated with smoking habits [39,40,41,42]. Additionally, the combination of inappropriate dietary habits with smoking has been considered an increased risk factor for lung cancer [43]. For these reasons, smoke-free policies associated with proper food literacy to improve eating patterns are of the utmost importance, especially in early adulthood, as a way to influence habits for the rest of adult life.

One of the other aspects investigated is related to alcohol consumption, which is believed to be high among university students as a result of social interactions, parties and academic festivities [44,45,46]. The results obtained showed that most participants (nearly two-thirds) consume alcohol regularly. A longitudinal study by Tarrant et al. [47] among United Kingdom university students showed that about half of them are categorized as high risk for alcohol use, with consumption patterns greatly motivated by group influences and expectations, and prevailing over time. Zhu et al. [48] suggest that family economic hardship may be associated with university students’ increased risk of alcohol consumption, primarily due to perceived discrimination and impulsivity. Benka [46] explored alcohol use among university students and reported that autonomous self-regulation of alcohol consumption led to a lower incidence of alcohol use, and that it was partially mediated by social, coping and mood enhancement motivations. Lemma et al. [44] observed that being male and having intimate friends who consume alcohol were among the factors that boost the risk of alcohol abuse disorders. Our results, also supported by the literature, indicate a high prevalence of alcohol consumption among university students. Nevertheless, contrary to tobacco regulations, which are very strict in Portugal, the control over alcohol sales is only focused on underage individuals, and even for those aged under 18 years, it is possible to buy alcohol in some small shops, which are less controlled. In the case of university students, there are no limitations, except inside the university campuses, where it is not possible to buy alcohol, and right outside the campus, where it is possible to find many bars and pubs with alcohol, especially for consumption after classes and in the night when the students gather for social engagement. Therefore, the recommendations for professionals and policymakers would be to adopt stricter regulations for alcohol than those that have been put into practice for smoking in Portugal in the past years.

The adoption of proper food habits, aimed at preserving health and wellbeing, is much dependent on knowledge, and for that reason, investigating food literacy is an essential tool to study the problem of incorrect eating habits and its consequent effect on disease, as well as to help define policies to improve knowledge about food consumption and health. Food literacy can be defined as a set of cognitive and social skills associated with the ability to acquire and understand information about food and nutrition to make appropriate nutritional decisions [49]. According to Malan et al. [50], food literacy is influenced by external factors that shape personal development and the application of the knowledge, skills and behaviours required for healthy eating. Food literacy among university students is still poorly understood, although higher education is recognized as a unique opportunity for promoting food literacy. A study by Rhea et al. [51] evaluated the factors associated with food literacy among university students, and found those factors to be health and nutrition, taste, food preparation, planning and decision-making, and convenience. A study about the level of food literacy and how this relates to food intake and happiness was conducted among Seoul citizens [52], which identified some vulnerable groups in terms of food literacy as being under thirty years old, of male sex and students. The study also showed how food literacy improved happiness and food-related life satisfaction. For rural young populations, there is the potential to enhance food literacy in a scholar context, and effective promotion of food literacy must be based on a holistic approach considering the roles of all relevant stakeholders [53].

Food literacy was assessed in this study in three complementary dimensions: ‘Literacy about food nutritional value’ and composition, ‘Literacy about labelling and food choice’ and ‘Literacy about healthy eating practices’. With respect to the first dimension (‘Literacy about food nutritional value’), if an individual is aware of the nutritive benefits of consuming certain food items and what health benefits these bring, he/she can be more motivated to consume these nutritive foods in detriment of other less healthy foods [54,55,56]. Nutrition education has been reported as associated with dietary patterns changing and health status improvement, particularly in some non-communicable diseases like type 2 diabetes or cardiovascular diseases, among others, causing a huge burden for society [56,57,58,59,60,61,62]. In what concerns the second dimension (‘Literacy about labelling and food choice’), the information provided on food labels is pivotal for the promotion of healthy eating, and that is why they are included in the domains of food literacy analysed in this study. For those with lower food literacy, tools such as the Nutri-Score or the Nutritional Traffic Light provide easy access to information that helps them make better food choices. Hoge et al. [63] studied a sample of university students in Belgium and concluded that the Nutri-Score is helpful to guide students in their food choices. A study conducted in Portugal by Silva et al. [64] showed that consumers usually read the food labels and recognize their importance, but fail to fully understand all the information contained therein. Finally, regarding the third dimension (‘Literacy about healthy eating practices’), the information about healthy eating and what is better to improve one’s health status is also a fundamental baseline to start practicing a healthier diet or to maintain appropriate dietary habits if that is already the case. Additionally, the development of science in the fields of health and nutrition is advancing very fast, and new trends and recommendations are made available on a regular basis, sometimes even contradicting previous perceptions or guidelines. One such example is the role of fats in health and disease. Fats have been considered to increase the risk factors for cardiovascular diseases, and, for that reason, they have been considered a food to avoid. However, new evidence has been released that contradicts this to some extent, by evidencing the different roles of saturated and unsaturated fatty acids (FA). In the review by Schwingshackl et al. [65], findings from systematic reviews of prospective observational studies revealed no association between total fat, saturated or unsaturated fatty acids, and the risk of non-communicable diseases. Nevertheless, some evidence supports the recommendation of replacing saturated FA with monounsaturated and polyunsaturated FA and avoiding the consumption of industrial total FA. Hence, updated information about food, nutrition and health is necessary to keep the guidelines and recommendations appropriate to improve public health.

When evaluating the possible influence of sociodemographic or academic variables on food literacy, it was found that food literacy does not vary significantly between female and male participants. Although not statistically significant, some differences were encountered between sexes, namely that females are more literate about the nutritional composition of foods and healthy eating practices than males, and, on the contrary, males are more literate in labelling and food choices. The work by Palumbo et al. [66] also reported that sex was not found to be related to food literacy in Italian women and men, although women were found to have a better ability to plan and manage food consumption. However, Gartaula et al. [53] reported that food literacy was higher for male than female students at academic and research institutions in Nepal.

When analysing the level of food literacy according to nationality, highly significant statistical differences were found. Nationality and social environments have been demonstrated to influence food literacy. Luque et al. [67] validated the self-perceived food literacy of a sample of Spanish university students, and obtained a five-factor scale, contrary to the seven-factor structure of the initial scale, which had been validated previously in an adult sample in the Netherlands [68]. The development and validation of the Food and Nutrition Literacy Questionnaire for Chinese adults [69] also revealed a different scale, composed of four dimensions: one was knowledge and three were practical (ability of selection, preparing food and eating). In the same study [69], the authors found that sociodemographic factors were predictors of food literacy. Another study conducted in China [70] revealed that providing nutrition education for parents can result in better food literacy and healthier eating among children. The study by Murakami et al. [71] carried out on a sample of Japanese adults revealed that females had higher nutrition knowledge than males, and those aged over sixty years had lower nutrition knowledge. Differences in food literacy according to nationality result from different social and political contexts, diverse educational systems and even cultural beliefs and gastronomic traditions. Portugal was one of the seven countries that subscribed for the recognition of the Mediterranean Diet (MD) as World Cultural Heritage, and as a result of that, the MD was officially inscribed in 2013 on the Representative List of the Intangible Cultural Heritage of Humanity of UNESCO. The MD is not only a dietary pattern but also comprises some social and lifestyle aspects to compose a healthy mode of eating [72]. The MD has been considered a healthy dietary pattern [73,74,75,76] and has been recommended as a pattern to follow, especially but not only among the southern European or northern African countries around the Mediterranean Sea. However, the influence of nationality on food literacy observed in this work must be interpreted on a limited scale, given that the number of foreign participants was much reduced as compared with the Portuguese ones. In this way, results cannot be fully generalized. Still, they give some hints about the possible differences between university national students and those from abroad, and also function as a way to use the university to spread information about the MD and implement in the foreign students the desire to adopt this pattern and to disseminate it, taking it back to their countries of origin.

The results of this research revealed that there are no significant differences in the food literacy of the students attending different academic degrees. Nevertheless, some trends were observed, namely that the students attending a License degree are those with greater literacy about the nutritional composition of foods and about healthy eating practices, while master’s students showed the highest literacy on labelling and food choices.

Palumbo et al. [77] suggest that food literacy is high in Italy, especially among the elderly, people with low education, and those suffering from financial deprivation, which are more likely to present lower levels of food literacy. The determinants of poor dietary habits are complex, so improving eating behaviour requires an interdisciplinary approach that acknowledges the social context [78]. Academic performance is very much linked with socioeconomic environments, and it is also known that nutrition is one of the most important and modifiable environmental factors that may affect brain development and, consequently, cognition and academic performance [79,80]. Better nutrition status must therefore be promoted in order to enhance academic achievements, and the other way around is also valid, i.e., higher nutrition knowledge and food literacy might be expected among students with better academic performance [81].

It was further observed in this study that students who do not consume alcohol are the group with the highest literacy about healthy eating practices and also with the highest global food literacy. Alcohol consumption tends to be characteristic of people less interested in healthy habits, and therefore, those who do not consume alcoholic beverages also tend to pay more attention to which foods they consume. Healthy food and beverage consumption are usually linked and constitute a concern for those who want to use food and drink as a way to improve global health status and well-being. Tobacco and alcohol consumption are among the most common unhealthy lifestyles among university students. The study by Zamboanga et al. [82] used the Brief Young Adult Alcohol Consequences Questionnaire to examine the effects of alcohol abuse in university students from five countries—Australia, New Zealand, Canada, Argentina and the United States—and confirmed that these problems are common to academic contexts in different countries. Peretti-Watel et al. [83] reported that for French students over a period of over 10 years, the frequencies of beer consumption and alcohol intoxication greatly increased, especially among females. However, Gawor et al. [84] found that moderate physical exercise can contribute to reducing alcohol cravings among United Kingdom university students. Tobacco use continues to pose serious public health risks, being considered a major cause of preventable diseases and deaths [35,85]. Early start increases the risk of continued regular smoking habits. Early adulthood is, therefore, frequently associated with increased smoking and the establishment of regular smoking habits. This may also contribute to the academic environment by favouring tobacco use or initiation through experimentation incentives by smoking peers [86,87,88].

The results from this work in global health highlighted some possible strategies to promote the dissemination of information and the increase in food literacy as tools to improve eating habits and healthy status among university populations in Portugal. Promotion of training sessions, practical activities, university competitions and/or gamification can be an effective tool to improve food literacy at this stage of life, with a positive impact on the health of those adults in the future. Planning differentiated actions according to sex does not seem necessary, since the levels of food literacy are similar for men and women, but nationality was found to be a major factor in the variability of food literacy. In this way, specific actions could be implemented to improve the food literacy of foreign incoming students at the Portuguese universities, promoting healthy eating patterns like those of the MD.

## 5. Conclusions and Limitations

The study carried out focused on food literacy among university students in Portugal. Regarding sociodemographic variables, it was found that the majority of students, mostly females, have a moderate level of literacy. Even so, about a quarter of the sample has low levels of food literacy.

In what concerns food literacy among groups, university students of Portuguese nationality showed higher levels of literacy when compared to students from abroad, but no significant differences were found for age, sex, academic performance or lifestyle variables. Nevertheless, some variations were encountered, namely, that (a) literacy about food nutrition value and composition is higher for female students, aged between 20 and 21 years, with very good academic performance, who smoke and consume alcohol; (b) literacy about labelling and food choice is higher for male students, aged 22 years or over, with very good academic performance, who never smoked but who consume alcohol; (c) literacy about healthy eating practices is higher for female students, with excellent academic performance, who smoke but do not consume alcohol.

Following the conclusions, we would like to point out some limitations of this study. It should be noted that data collection was carried out through the application of online questionnaires, without the presence of the researcher to clarify possible doubts raised at the moment of responding to the questionnaire. Additionally, there is a chance that some answers given may not have been “real”, as some participants may have given what they believe would be the “right” answer instead of the true one. It is important to mention the size of the sample, which, despite being reasonable in number, is not fully representative of university students at the national level, thus not allowing a true generalization of the results obtained. Additionally, the group sizes are quite uneven regarding some characteristics, like the number of international students or the number of older students. This results from the natural heterogeneity of the university populations in Portugal. There is a lower number of older students, who are frequenting universities as lifelong learners or as a way to move in a different professional direction. Additionally, there is a low number of international students, coming through international programs such as ERASMUS, as compared to national students. Still, these groups, although minority, in fact describe the reality of the Portuguese Higher Education system, and for that reason, these groups, even though minority, were invaded in the study for providing additional information. Naturally, these particular results for minority groups are much more limited in their interpretation and do not allow generalization, and that is a limitation. The option of excluding the minority groups and using only standard students was discarded because, even though they are minority groups, these can give some trends that are of interest to fully understand the food literacy of the university population in Portugal.

Framing the empirical study as a type of cross-sectional correlational analytical research, it is considered that these assumptions also constitute a methodological limitation, since the evaluations were carried out in a single “moment”, with no follow-up period for the participants. However, this type of study has advantages, such as, for example, being faster, less expensive, less complex in logistic terms, and not subject to problems such as loss of follow-up (as is the case with longitudinal studies).

Despite the limitations, we believe the results obtained increased knowledge about the present status of food literacy among the university students in Portugal, which can be helpful to reinforce measures to promote even higher literacy with positive outcomes concerning the eating habits and general health status of the students, which may endure into adulthood.

## Figures and Tables

**Table 1:** Analysis of lifestyle variables according to sex.

Variables	Female Sex		Male Sex		Total		Adjusted Residuals		Chi-Square Test ^1^	
	N 736	% 79.7	N 188	% 20.3	N 924	% 100	Fem.	Mal.	Stat	*p*
**Tobacco consumption**									3.54	0.170
I presently smoke	85	9.2	31	3.4	116	12.6	−1.8	1.8		
I used to smoke, but stopped	94	10.2	25	2.7	119	12.9	−0.2	0.2		
I never smoked	557	60.3	132	14.3	689	74.6	1.5	−1.5		
**Alcohol consumption**									7.35	0.025
Yes	533	57.7	150	16.2	683	73.9	−2.1	2.1		
No	140	15.2	20	2.2	160	17.3	2.7	−2.7		
Never drunk	63	6.8	18	1.9	81	8.8	−0.4	0.4		

^1^ Stat = Test statistics; *p* = significance (two-tailed), significant for *p* < 0.05.

**Table 2:** Statistics related to food literacy indices in university students.

	N	Min	Max	Mean	SD	CV (%)	Sk/err	K/err
**Dimension 1** Literacy about food nutritional value and composition	10	25	100	80.41	14.88	18.51	−6.11	−0.30
**Dimension 2** Literacy about labelling and food choice	7	25	100	77.94	16.39	21.03	−6.20	−0.70
**Dimension 3** Literacy about healthy eating practices	9	25	100	81.79	13.85	16.93	−8.38	4.30
**Global** **Food literacy**	26	25	100	80.22	13.84	17.25	−6.14	1.34

N = number of items, Min = minimum value, SD = standard deviation, CV = coefficient of variation, Sk/err = skewness/error, K/err = kurtosis/error.

**Table 3:** Level of food literacy according to sex and nationality.

Variable	Group		Food Literacy Level			Chi-Square Test ^1^	
			Low	Medium	High	Stat.	*p*
Sex	Female (N = 736)	N (%)	185 (20.0%)	361 (39.1%)	190 (20.6%)	1.12	0.571
	Male (N = 188)	N (%)	52 (5.6%)	94 (10.1%)	42 (4.5%)		
	Total (N = 924)	N (%)	237 (25.6%)	455 (49.2%)	232 (25.1%)		
	Adjusted residuals	Female	−0.7	−0.2	1.0		
		Male	0.7	0.2	−1.0		
Nationality	Portuguese (N = 887)	N (%)	218 (23.6%)	443 (47.9%)	226 (24.5%)	13.352	0.001
	Other (N = 37)	N (%)	19 (2.1%)	12 (1.3%)	6 (0.6%)		
	Total (N = 924)	N (%)	237 (25.6%)	455 (49.2%)	232 (25.1%)		
	Adjusted residuals	Port.	−3.7	2.1	1.3		
		Other	3.7	−2.1	−1.3		

^1^ Stat = Test statistics; *p* = significance (two-tailed), significant for *p* < 0.05.

**Table 4:** Mann–Whitney test to analyse the relationship between food literacy dimensions and sex and nationality.

Variable	Group ^1^	Food Literacy ^2^			
		D1	D2	D3	Global
Sex	Female OR (N = 736)	467.74	460.31	469.09	466.55
	Male OR (N = 188)	441.98	471.06	436.70	446.64
	U Mann–Whitney test (Z, *p*) ^3^	Z = −1.187 *p* = 0.235	Z = −0.496 *p* = 0.620	Z = −1.493 *p* = 0.136	Z = −0.914 *p* = 0.361
Nationality	Portuguese OR (N = 887)	467.52	466.96	466.96	467.46
	Other OR (N = 37)	334.16	367.84	355.49	343.68
	U Mann–Whitney test (Z, *p*) ^3^	Z = −2.814 *p* = 0.005	Z = −2.215 *p* = 0.027	Z = −2.502 *p* = 0.012	Z = −2.767 *p* = 0.006

^1^ OR = Odds ratio. ^2^ D1 = Dimension 1—Literacy about food nutritional value and composition; D2 = Dimension 2—Literacy about labelling and food choice; D3 = Dimension 3—Literacy about healthy eating practices. ^3^ Z = Test statistics; *p* = significance (two-tailed), significant for *p* < 0.05.

**Table 5:** One-way analysis of variance (ANOVA) of the relationship between food literacy dimensions and age.

Food Literacy	Age Groups			ANOVA ^2^	
	≤19 Years (N = 313)	20–21 Years (N = 295)	≥22 Years (N = 316)		
	M ± SD ^1^	M ± SD ^1^	M ± SD ^1^	F	*p*
**Dimension 1** Literacy about food nutritional value and composition	79.80 ± 3.96	80.26 ± 15.66	81.41 ± 14.87	0.673	0.510
**Dimension 2** Literacy about labelling and food choice	77.33 ± 15.52	77.31 ± 17.47	79.13 ± 16.39	1.261	0.284
**Dimension 3** Literacy about healthy eating practices	81.85 ± 12.99	81.89 ± 14.19	81.79 ± 13.85	0.028	0.972
**Global** **Food literacy**	79.84 ± 12.80	80.03 ± 14.67	80.77 ± 13.84	0.399	0.671

^1^ M = Mean value, SD = Standard deviation. ^2^ F = test statistic, *p* = significance (*p* < 0.05).

**Table 6:** One-way analysis of variance (ANOVA) of the relationship between food literacy dimensions and the type of course frequented.

Food Literacy	Type of Course ^1^				ANOVA ^2^	
	CTESP (N = 19)	License (N = 693)	Master (N = 161)	Other (N = 51)		
	M ± SD	M ± SD	M ± SD	M ± SD	F	*p*
**Dimension 1** Literacy about food nutritional value and composition	77.6 ± 15.6	80.7 ± 14.6	79.7 ± 16.2	79.2 ± 13.4	0.560	0.642
**Dimension 2** Literacy about labelling and food choice	73.9 ± 18.3	78.0 ± 16.1	78.0 ± 17.8	78.0 ± 15.7	0.379	0.755
**Dimension 3** Literacy about healthy eating practices	77.3 ± 16.3	82.2 ± 13.4	80.5 ± 15.4	81.9 ± 13.3	1.296	0.275
**Global** **Food literacy**	7 7.3 ± 16.3	81.2 ± 13.2	80.3 ± 15.4	80.9 ± 12.7	0.702	0.551

^1^ CTESP = Higher Technical Professional Course; M = Mean value, SD = Standard deviation. ^2^ F = test statistic, *p* = significance (*p* < 0.05).

**Table 7:** Level of food literacy according to self-reported academic performance.

Academic Performance		Food Literacy Level			Chi-Square Test ^1^	
		Low	Medium	High	Stat.	*p*
Poor (N = 8)	N (%)	2 (0.2%)	5 (0.5%)	1 (0.1%)	12.954	0.113
Reasonable (N = 164)	N (%)	47 (5.1%)	87 (9.4%)	30 (3.2%)		
Good (N = 560)	N (%)	145 (15.7%)	266 (28.8%)	149 (16.1%)		
Very good (N = 174)	N (%)	42 (4.5%)	83 (9.0%)	49 (5.3%)		
Excellent (N = 18)	N (%)	1 (0.1%)	14 (1.5%)	3 (0.3%)		
Total (N = 924)	N (%)	237 (25.6%)	455 (49.2%)	232 (25.1%)		
Adjusted residuals	Poor	0.0	0.8	−0.8		
	Reasonable	1.0	1.1	−2.2		
	Good	2.0	−1.3	1.3		
	Very good	−0.5	−0.5	1.0		
	Excellent	−2.0	2.4	−0.8		

^1^ Stat = Test statistics; *p* = significance (two-tailed), significant for *p* < 0.05.

**Table 8:** Kruskal–Wallis test to analyse the relationship between food literacy dimensions and self-reported academic performance and average classification.

Variable	Group ^1^	Food Literacy ^2^			
		D1	D2	D3	Global
Self-reported academic performance	Poor OR (N = 8)	298.06	406.00	416.06	354.25
	Reasonable OR (N = 164)	422.68	434.08	432.83	429.15
	Good OR (N = 560)	466.06	462.29	461.00	462.83
	Very Good OR (N = 174)	495.11	492.91	493.84	495.54
	Excellent OR (N = 18)	472.31	459.06	497.08	485.00
	Kruskal–Wallis test (H, *p*) ^3^	H = 9.506 *p* = 0.050	H = 4.534 *p* = 0.339	H = 5.038 *p* = 0.283	H = 6.686 *p* = 0.153
Average classification	<10 points OR (N = 5)	382.00	520.90	519.70	470.20
	10–13 points OR (N = 268)	426.40	443.14	434.99	434.15
	14–16 points OR (N = 551)	476.03	459.45	469.96	468.63
	17–20 points OR (N = 91)	466.05	489.53	449.96	463.23
	Kruskal–Wallis y test (H, *p*) ^3^	H = 7.066 *p* = 0.070	H = 2.471 *p* = 0.481	H = 3.552 *p* = 0.314	H = 3.126 *p* = 0.373

^1^ OR = Odds ratio. ^2^ D1 = Dimension 1—Literacy about food nutritional value and composition; D2 = Dimension 2—Literacy about labelling and food choice; D3 = Dimension 3—Literacy about healthy eating practices. ^3^ H = Test statistics; *p* = significance (two-tailed), significant for *p* < 0.05.

**Table 9:** Kruskal–Wallis test to analyse the relationship between food literacy dimensions and self-reported smoking and alcohol consumption habits.

Variable	Group ^1^	Food Literacy ^2^			
		D1	D2	D3	Global
Smoking	I presently smoke OR (N = 116)	493.13	459.28	472.01	479.34
	I used to, but stopped OR (N = 119)	412.08	417.22	411.79	412.11
	I never smoked OR (N = 689)	466.05	470.86	469.66	468.37
	Kruskal–Wallis test (H, *p*) ^3^	H = 5.957 *p* = 0.051	H = 4.186 *p* = 0.124	H = 4.988 *p* = 0.083	H = 5.048 *p* = 0.080
Alcohol consumption	Yes OR (N = 683)	466.19	466.47	464.87	465.62
	No OR (N = 160)	466.10	464.24	466.62	466.63
	Never drunk OR (N = 81)	424.28	425.61	434.42	428.07
	Kruskal–Wallis y test (H, *p*) ^3^	H = 1.839 *p* = 0.399	H = 1.762 *p* = 0.422	H = 0.998 *p* = 0.607	H = 1.482 *p* = 0.477

^1^ OR = Odds ratio. ^2^ D1 = Dimension 1—Literacy about food nutritional value and composition; D2 = Dimension 2—Literacy about labelling and food choice; D3 = Dimension 3—Literacy about healthy eating practices. ^3^ H = Test statistics; *p* = significance (two- tailed), significant for *p* < 0.05.

## Data Availability

Data are available from the corresponding author upon request.

## Appendix A

In this section, the questions used in the survey are listed.

1. **Sociodemographic and behavioural characterization of the sample**

**Sociodemographic data:**
**Age**: ______ years
**Sex**: ▯ Male ▯ Female
**Nationality**: ▯ Portugal ▯ Abroad (ex. Erasmus student)
**Academic data:**
**University course frequented**: __________________
▯ CTESP (Higher Technical Professional Course) ▯ Bachelor ▯ Master ▯ Other
**Course year**: __________
**How do you consider your academic performance?**
▯ Excellent ▯ Very good ▯ Good ▯ Reasonable ▯ Bad
**Average course classification up to the present moment (scale from 0 to 20 points, pass = 10 points):**
▯ <10 points ▯ 10–13 points ▯ 14–16 points ▯ 17–20 points
**Smoking habits:**
**Regarding the consumption of cigarettes, cigars, pipes and/or electronic cigarettes, which of the following conditions applies to you?**
▯ I currently smoke ▯ I used to smoke, but I have stopped ▯ I never smoked
**How often do you smoke?**
▯ Occasionally ▯ Every day ▯ _____cigarettes per day
**Drinking habits:**
**During the last 12 months, have you consumed alcoholic beverages (beer, wine, spirits, cider or others)?**
▯ Yes ▯ No ▯ Never did
**How many times in the last 12 months have you consumed three or more alcoholic drinks in a day?**
▯ Several times a week ▯ Once a week ▯ Once a month ▯ Less than once a month ▯ Never
**During the last 30 days, did you drink any of these alcoholic beverages (beer, wine, spirits, cider or other)?**
▯ Yes  ▯ No

2. **Items used to measure food literacy**

Classify each of the following statements about food using a 5-point scale (1 = very difficult, 2 = difficult, 3 = easy, 4 = very easy, 5 = I don’t know).

(a) **Literacy about food nutritional value and composition**
  - Understand the information contained in the Portuguese Food Wheel.
  - Find information on the nutritional quality of beverages.
  - Use the information to match your daily fluid intake needs.
  - Understand the usefulness of taking food supplements (multivitamins, vitamins, calcium, Omega 3, etc.).
  - Find information on the differences between saturated and unsaturated fats.
  - Understand the effects resulting from the consumption of saturated and unsaturated fats.
  - Understand the type of carbohydrates you eat in your diet.
  - Understand the benefits or drawbacks of excessive consumption of dietary fibre.
  - Understand the recommended amounts for protein intake.
  - Moderate your protein intake.

(b) **Literacy about labelling and food choice**
  - Find information on how to interpret food labels.
  - Find information about the nutritional semaphore on food labels.
  - Understand the nutritional semaphore on food labels.
  - Use food labelling to help make healthier food choices.
  - Understand the recommendations on the amounts of food that should be consumed, when presented in mass (grams).
  - Find information about the Mediterranean Diet.
  - Practice eating habits that conform to the standards of the Mediterranean Diet.

(c) **Literacy about healthy eating practices**
  - Find information on healthy eating (website of the General Health Directory (DGS)—official Portuguese website).
  - Find information on healthy eating (internet, television, books/magazines).
  - Understand information on healthy eating (DGS website).
  - Find information on daily meal frequency.
  - Understand the importance of eating several times a day.
  - Find information on diets and regimens (calorie restriction; vegetarian/vegan; organic; diets suitable for certain diseases/intolerances (e.g., gluten-free).
  - Understand the information about diets and regimens found on the internet.
  - Find information on recommended portion sizes for each type of food.
  - Understand recommended consumption dosages by food groups when expressed in portions.
